# Evolution of knowledge on ovarian physiology and its contribution to the widespread application of reproductive biotechnologies in South American cattle

**DOI:** 10.21451/1984-3143-AR2018-0007

**Published:** 2018-08-03

**Authors:** Reuben J. Mapletoft, Gabriel A. Bó, Pietro S. Baruselli, Alejo Menchaca, Roberto Sartori

**Affiliations:** 1 WCVM, University of Saskatchewan, Saskatoon, SK, Canada; 2 Instituto de Reproducción Animal Córdoba (IRAC) and Universidad Nacional de Villa Maria, Cordoba, Argentina; 3 Department of Animal Reproduction, University of São Paulo, São Paulo, SP, Brazil; 4 Instituto de Reproducción Animal Uruguay Fundación IRAUy, Montevideo, Uruguay; 5 Department of Animal Science, University of São Paulo, Piracicaba, SP, Brazil

**Keywords:** AI, embryo transfer, estrus synchronization, follicular wave, superovulation.

## Abstract

As our understanding of ovarian function in cattle has improved, our ability to control it has also increased. Luteal function in cattle has been studied in detail, and prostaglandin F2α has been used for several years for the elective induction of luteal regression. More recently, follicle wave dynamics has been studied and protocols designed to induce follicular wave emergence and ovulation have reduced, and even eliminated, the need for estrus detection. The addition of progestin-releasing devices, estradiol, GnRH and equine chorionic gonadotropin (eCG) have provided opportunities for fixed-time AI (FTAI) and possibilities for increased pregnancy rates. In embryo transfer programs, these same treatments have eliminated the need for estrus detection, permitting fixed-time embryo transfer and the initiation of superstimulatory treatments without regard to day of the estrous cycle. Collectively, new treatment protocols have facilitated the application of assisted reproductive technologies, and this is especially true in South America. Over the last 20 years, the use of AI in South America has increased, due largely to the use of FTAI. There has been more than a 10-fold increase in the use of FTAI in Brazil with more than 11 million treatments in 2016, representing 85% of all AI. Similar trends are occurring in Argentina and Uruguay. Production of *in vivo-*derived (IVD) embryos has remained relatively stable over the years, but *in vitro* embryo production (IVP) has increased dramatically over the past 10 to 15 years, especially in Brazil where more than 300,000 IVP embryos were produced in 2010. World-wide, more than 666,000 bovine IVP embryos were produced in 2016, of which more than 57% were produced in South America. The use of assisted reproductive technologies has facilitated the dissemination of improved genetics and increased reproductive performance; other South American countries are now following suit.

## Introduction

Increasing knowledge of ovarian physiology in cattle over the past 50 years has provided approaches for the manipulation of ovarian function. Protocols designed to control both luteal and follicular function have improved estrus synchronization and permitted fixed-time AI (FTAI) and fixed-time embryo transfer (FTET), and the initiation of superstimulatory treatments at a self-appointed time. Bovine practitioners around the world are now using these reproductive technologies, and this is especially the case in South America. This review will briefly describe improvements in our understanding of ovarian physiology in cattle and discuss how this has affected the application of assisted reproductive biotechnologies in South America, with special emphasis on Brazil, Argentina and Uruguay where much of the research and practical application is taking place.

## A brief review of recent knowledge on ovarian physiology in cattle

Ovarian follicles in cattle grow in a wave-like fashion. A follicular wave consists of the synchronous emergence of a group of antral follicles 4 to 5 mm in diameter followed in 2 or 3 days by the selection of one follicle to become dominant, while subordinates become atretic (Ginther *et al*., 1989a). Estrous cycles in cattle are composed of either 2 or 3 follicular waves (Ginther *et al*., 1989b), although 4-wave cycles have been reported in *B. indicus* breeds (Bó *et al*., 2003). In both 2- and 3-wave cycles, emergence of the first follicular wave occurs on the day of ovulation (Day 0). Because of the presence of the mid-cycle CL, the dominant follicle of the first wave regresses, and a second wave emerges on Days 9 or 10 in 2-wave cycles, and Days 8 or 9 in 3-wave cycles, with the third wave emerging on Days 15 or 16. Follicular waves occur in heifers before puberty and in postpartum cows, before the first ovulation (reviewed in Adams, 1999).

Recruitment of follicular waves and selection of the dominant follicle are based on differential responsiveness of antral follicles to FSH and LH (reviewed in Adams, 1999). Surges in circulating FSH are followed in 1 to 2 days by the appearance of a group of follicles that are 4 to 5 mm in diameter. FSH is then suppressed by estradiol and inhibin produced by follicles in the wave (especially the future dominant follicle), leading to the selection of a dominant follicle approximately 3 days after wave emergence. The dominant follicle acquires more LH receptors on its granulosa cells than its subordinates and is able to shift its gonadotropin dependence to LH during the period of low FSH; it continues to grow while subordinates requiring FSH regress (Ginther *et al*., 2001). Suppression of LH by progesterone from the CL causes the dominant follicle of the first wave (and of the second wave in 3- wave cycles) to eventually cease its metabolic functions and regress, which leads to an FSH surge and emergence of a new follicular wave (Adams, 1999). Luteal regression results in increased LH pulse frequency, increased growth of the dominant follicle, elevated estradiol concentrations, an LH surge and ovulation.

The number of follicular waves in cattle depends on the duration of follicular dominance in the first wave; it is 3 days longer and the onset of regression occurs later during 2-wave cycles than 3-wave cycles. However, the onset of luteolysis occurs earlier in 2- wave cycles resulting in an interovulatory interval of 20 days, as compared to 23 days in 3-wave cycles (Adams, 1999; Sartori *et al*., 2004). The reason for difference in the duration of follicular dominance in the first wave between wave types is unknown. The dominant follicle present at the time of luteolysis becomes the ovulatory follicle, and emergence of the next wave is delayed until the ensuing ovulation (Kastelic and Ginther, 1991).

**Figure 1:**
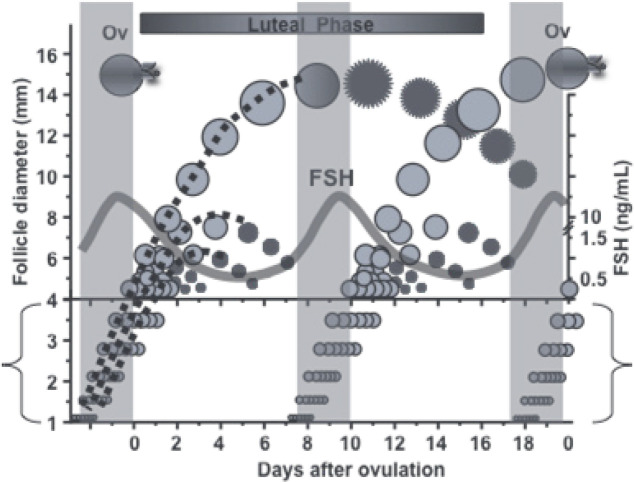
A two-wave ovarian follicular wave pattern detected in follicles as small as 1 mm in diameter. Small follicles (1 to 3 mm) in parentheses illustrate wave emergence 2.5 days earlier than previously detected (i.e., at 4 to 5 mm). Note that the growth rate of the follicle destined to become dominant (dotted line) is similar to others in the wave until about 5 days after wave emergence at 1 mm, and has a size advantage over those destined to become subordinate as small as 1 mm in size (Adams et al., 2008). Compliments of GP Adams.

Follicular wave dynamics as described has been limited to follic≥les 4 mm because of the resolution of early ultrasound equipment. However, newer equipment has permitted the identification of the future dominant follicle at a diameter of 1 mm, suggesting that 1 to 3 mm follicles also develop in a wave-like manner (Fig. 1). Adams *et al*. (2008) pointed out that at a microscopic level, there is no morphologic distinction between follicles that are 1 to 3 mm and those that are more than 3 mm, and that at a cellular level, both size categories not only express FSH receptors but have a similar level of expression on a per granulosa cell basis (reviewed in Bao and Garvarick, 1998). They also provide evidence that these small antral follicles respond to transient elevations in circulating FSH and that their growth progresses over a period encompassing the entire FSH surge. Thus, 1 to 3 mm follicles may be an important component of the wave. This has implications for the optimization of superstimulation for both *in vivo* and *in vitro* embryo production in cattle (Garcia Guerra *et al*., 2015).

There are several differences in reproductive function between *B. indicus* and *B. taurus* breeds of cattle and these must be considered when designing assisted reproductive programs. Nutrition, post-partum anestrus and age of onset of puberty are especially important in *B. indicus* cattle. They also have a shorter estrus period, often expressed during the night, and although follicular dominance is similar, maximum diameters of the dominant follicle and CL are smaller (Bó *et al*., 2003), as are dominant follicle diameters at the time of selection and ovulation (Sartori *et al*., 2001; Sartorelli *et al*., 2005; Gimenes *et al*., 2008). In addition, *B. indicus* breeds tend to be more sensitive to steroid hormones which must be considered in designing estrus synchronization programs (Bó *et al*., 2003). However, *B. indicus* breeds also have greater antral follicle counts (Batista *et al*., 2014; Sartori *et al*., 2016a) which has important implications for superstimulation and ovum pick-up and *in vitro* fertilization (OPU/IVF).

## Synchronization of estrus for artificial insemination or embryo transfer

### Prostaglandin F2α

Prostaglandin F2α (PGF) has become the most common treatment for elective induction of luteal regression and synchronization of estrus in cattle (reviewed in Odde, 1990). However, cattle must be cycling and PGF will not induce luteolysis during the first 5 days of the cycle (Seguin, 1987). In addition, the onset of estrus may occur over several days; treatment when the dominant follicle is near mature will result in ovulation in 2 or 3 days, whereas treatment when it is no longer viable will result in ovulation of the dominant follicle of the next wave, 4 to 5 days later (Kastelic *et al*., 1990). In a two-dose PGF synchronization scheme, an interval of 10 or 11 days has been recommended to ensure that all cattle have a PGF-responsive CL at the time of the second treatment. Although an interval of 11 days between PGF treatments has been found to be acceptable for heifers (Selk *et al*., 1988), higher conception rates have been reported in lactating dairy cows with a 14-day interval (Folman *et al*., 1990). Most other methods of estrus synchronization utilize PGF to regress an existing or new CL.

Acceptable pregnancy rates in embryo transfer are partially dependent upon the onset of estrus in the recipient being within 24-hour synchrony of the embryo donor or stage of development of the embryo (Bó *et al*., 2012). Pregnancy rates do not differ whether recipients are selected following detection of estrus in untreated animals or estrus synchronization. As estrus in donors will occur 36 to 48 hours after treatment with PGF, recipients must be treated 12 to 24 hours earlier than donors (Bó and Mapletoft, 2014).

### Progestins

Progesterone alters ovarian function in cattle by suppressing estrus and preventing ovulation, primarily by suppressing LH release. As progesterone does not suppress FSH secretion, follicle waves continue to emerge in the presence of a functional CL (Adams, 1999). Progestins given for intervals exceeding the lifespan of a CL result in synchronous estrus upon withdrawal, but fertility is usually low because exogenous progestins are generally less suppressive of LH than endogenous progesterone. The resulting high LH pulse frequency leads to a “persistent” follicle (Savio *et al*., 1993) with an oocyte that may be infertile (Revah and Butler, 1996).

Progesterone-releasing devices are now used to synchronize estrus in cattle (Mapletoft *et al*., 2003), and there are several such products available in Brazil and Argentina with different payloads of progesterone for different classes of cattle. Progestin devices are normally removed after 7 or 8 days and PGF is administered at that time (or 24 hours earlier); estrus occurs 48 to 72 hours later. Because of the short treatment period, persistent follicles do not occur and fertility following AI is usually normal. Progestin devices are well suited for the various approaches used to synchronize follicular development and ovulation (Mapletoft *et al*., 2003).

As protocols designed for estrus synchronization have depended on estrus detection, results are often disappointing because estrus detection is time consuming and inaccurate (Washburn *et al*., 2002). While acceptable conception rates may occur following estrus detection, pregnancy rates are low because of low submission rates. Estrus detection efficiency also affects the use of assisted reproductive technologies e.g., when PGF is used to synchronize cycling recipients in a 2-injection protocol, 80% are expected to show signs of estrus within 5 days of the second treatment. However, due to inefficient estrus detection, less than 50% receive an embryo (Bó *et al*., 2012). The problem may be greater if the recipients are *B. indicus* or *B. indicus* crosses under pasture conditions (Sartori and Barros, 2011). An alternative is to eliminate the need for estrus detection by applying fixed-time protocols (Baruselli *et al*., 2010; Bó *et al*., 2013).

## Manipulation of ovarian function for fixed-time AI or fixed-time embryo transfer

### Ultrasound-guided follicle ablation

**Figure 2:**
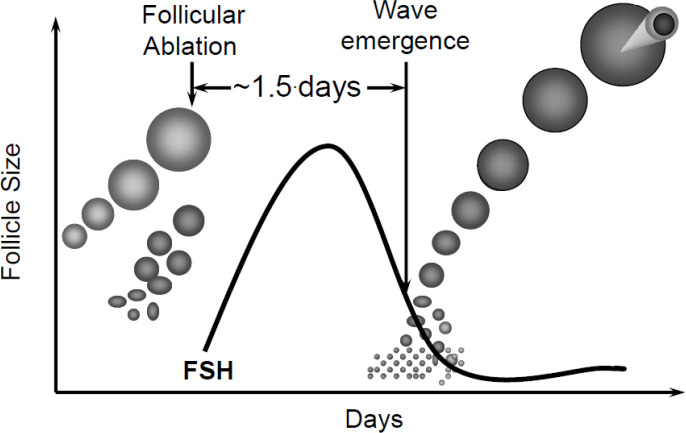
Synchronization of follicular wave emergence by ultrasound-guided follicle ablation. Aspiration of antral follicles causes the emergence of the next follicular wave by removing the suppressive effects of follicle products on FSH release. FSH surges and follicular wave emergence occurs 1 to 2 days later.

Transvaginal ultrasound-guided ablation of antral follicles results in emergence of a new follicular wave in approximately 1.5 days by removing the suppressive effects of follicle products (e.g., estradiol and inhibin) on FSH release (Bergfelt *et al*., 1994; Fig. 2). Although follicle ablation in combination with PGF is very efficacious in synchronizing follicle wave emergence and ovulation, it is not practical in the field.

### Estradiol and progesterone

**Figure 3:**
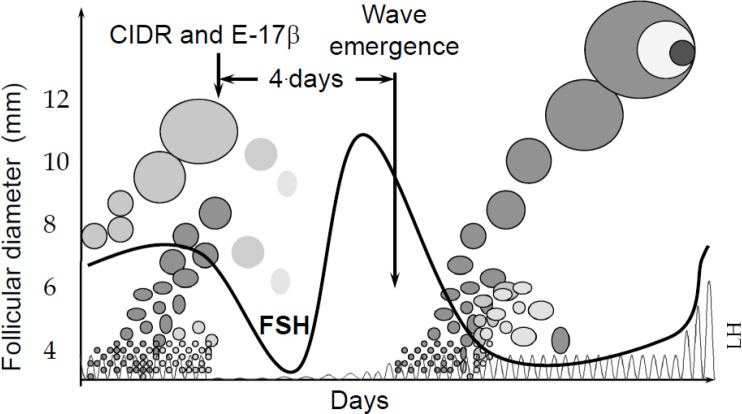
Synchronization of follicular wave emergence by estradiol/progestin treatment. Estradiol causes suppression of FSH and atresia of FSH-dependent follicles. Once the injected estradiol is metabolized, FSH surges and a new follicular wave emerges 3 to 5 days after treatment.

Estradiol valerate was originally used at the start of a 9-day progestin protocol to cause uterine- induced luteolysis (Wiltbank *et al*., 1965), but it has also been shown to suppress antral follicles (Bó *et al*., 1995). The mechanism of estrogen-induced follicle atresia appears to be systemic, and involves suppression of FSH (Fig. 3). Once exogenously administered estradiol is metabolized, FSH surges and a new follicular wave emerges. The administration of 2.5 to 5 mg estradiol- 17β (E-17β; reviewed in Bó *et al*., 2002) or 2 mg of estradiol benzoate (EB; Martinez *et al*., 2005) or estradiol valerate (EV; Colazo *et al*., 2005) in progestin- treated cattle results in emergence of a new follicular wave in 3 to 5 days.

In early estrus synchronization protocols, 2.5 mg EB is administered at the time of insertion of a progestin device which is removed 7 days later, at the time of administration of PGF (Mapletoft *et al*., 2003). A dose of 1 mg EB was given 24 hours later to induce an LH surge in 16 to 18 hours (Martinez *et al*., 2007) and ovulation approximately 24 hours later. This permitted FTAI with acceptable pregnancy rates. As an alternative, administration of 0.5 to 1.0 mg of estradiol cypionate (ECP; Colazo *et al*., 2003) at the time of progestin removal with FTAI 48 to 52 hours later is often used in South America (Sales *et al*., 2012: Martins *et al*., 2017). Pregnancy rates following FTAI have been shown to be improved in suckled beef cows and heifers, especially *B. indicus* crosses, when 300 to 400 IU of eCG is administered at the time of progestin removal (Baruselli *et al*., 2004; Bó *et al*., 2012; 2016). The administration of eCG stimulates dominant follicle growth and maturation, and increases progesterone production by the subsequent CL (*B. taurus*, Nunez-Olivera *et al*., 2014; *B. indicus*, Baruselli *et al*., 2004). Estradiol, progesterone and eCG treatments are also very useful for fixed-time embryo transfer (FTET) with a greater proportion of recipients selected for embryo transfer and higher pregnancy rates following transfer (Baruselli *et al*., 2010; Bó *et al*., 2012; Rodrigues *et al*., 2010).

### Gonadotropin releasing hormone (GnRH)

**Figure 4:**
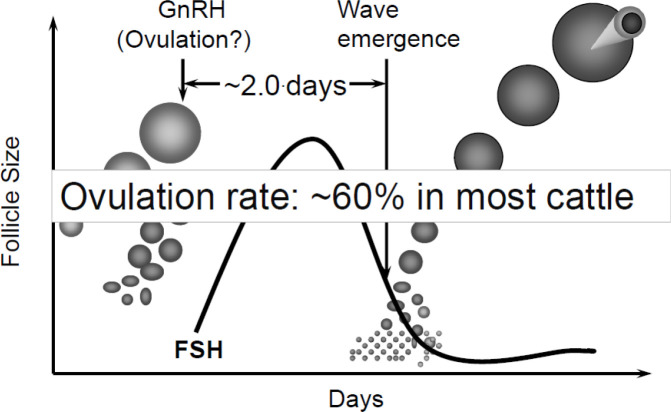
Synchronization of follicular wave emergence by GnRH treatment which causes ovulation of a growing dominant follicle. This removes the suppression of follicle products on FSH release; FSH surges and a new follicle wave emerges approximately 2 days after treatment. However, treatment at random stages of the estrous cycle results in less than 60% of animals ovulating and if ovulation does not occur, follicle wave emergence will not be synchronized.

GnRH treatment of cattle with a growing dominant follicle will induce ovulation and emergence of a new follicular wave 1 to 2 days later (Macmillan and Thatcher, 1991) by inducing LH release, but only when ovulation occurs (Martinez *et al*., 1999; Fig. 4). An ovulation synchronization protocol for FTAI in lactating dairy cattle utilizing GnRH has been developed by Pursley *et al*. (1995; Ovsynch). Administration of GnRH is followed in 7 days by PGF and a second GnRH in 48 hours, with FTAI 16 to 18 hours later. More recently, it has been shown that pregnancy per AI (P/AI) is improved in lactating dairy cattle if the interval from PGF to the second GnRH is increased to 56 hours (Ovsynch-56; Brusveen *et al*., 2008). The Ovsynch protocol has been more efficacious in lactating dairy cows than in heifers (Seguin, 1997). Although the cause is not clear, ovulation following the first GnRH has been reported to be higher in cows than heifers and a higher percentage of heifers show estrus early, resulting in reduced fertility following FTAI (Wiltbank, 1997).

Stage of development of the dominant follicle (Martinez *et al*., 1999), and stage of the estrous cycle (Vasconcelos *et al*., 1999; Moreira *et al*., 2000) affect response to the first GnRH. If the dominant follicle is immature or post-mature, ovulation may not occur and a new follicular wave will not emerge. Cattle respond most consistently between Days 5 and 12 of the estrous cycle, so a 2-injection PGF pre-synchronization treatment is often used before administration of the first GnRH (Moreira *et al*., 2001). Various other presynchronization protocols have been developed to improve P/AI following the use of the Ovsynch protocol (Stevenson, 2011). Alternatively, a progestin insert at the time of the first GnRH has also improved P/AI in beef heifers (Martinez *et al*., 2002), beef cows (Lamb *et al*., 2001) and anovulatory dairy cows (Stevenson *et al*., 2006; Bisinotto *et al*., 2013). Treatment with eCG at the time of progestin removal has also improved pregnancy rates in non-cycling beef cows (Bó *et al*., 2016).

More recently, a 5-day GnRH-based, Co-Synch protocol has been developed for beef cattle in North America, with higher P/AI than obtained with the more traditional 7-day Co-Synch protocol (Bridges *et al*., 2008; reviewed in Day, 2015). The physiological basis of this protocol is to reduce the progestin device insertion period to 5 days, avoiding the development of persistent follicles in cattle not ovulating to the first GnRH, and then lengthening the proestrus period to 72 hours to allow for greater dominant follicle development and higher circulating estrogen levels prior to ovulation. However, two injections of PGF are required with this protocol to induce luteal regression in animals that ovulate to the first GnRH. Several subsequent studies have been designed to eliminate the need for extra animal handling associated with two injections of PGF, but results have been inconsistent, possibly because of the inconsistent response to the first GnRH in heifers. Rabaglino *et al*. (2010) found no difference in P/AI, whether one or two injections of PGF were administered, while Peterson *et al*. (2011) reported that heifers given two injections of PGF tended to have higher P/AI. Colazo and Ambrose (2011) eliminated the initial administration of GnRH in the 5- day protocol without adversely affecting fertility. However, in a large study, Lima *et al*. (2013) found that P/AI was greater in heifers that received GnRH at progestin insertion and two injections of PGF after progestin removal (on Days 5 and 6) than in heifers that did not receive the initial GnRH, whether they received one or two injections of PGF. More recently, Kasimanickam *et al*. (2014) reported that the initial GnRH increased P/AI in beef heifers, but not in dairy heifers, and that P/AI in dairy heifers did not differ whether they received one or two injections of PGF. Perhaps, it is simply a matter of statistical power. In Argentina, an estradiol-based protocol with shortened progestin exposure and a lengthened proestrus, named J- Synch, has been developed (reviewed in Bó *et al*., 2016). This protocol has the advantage that an initial GnRH treatment (and subsequently, two injections of PGF) is not required. Use of a 6-day J-Synch protocol has resulted in higher P/AI in heifers than the conventional 8- day estradiol-based protocol (Bó *et al*., 2016).

GnRH-based protocols are also efficacious in the synchronization of ovulation in recipients (Bó *et al*., 2012). In *B. indicus* x *B. taurus* crossbred heifers, the overall pregnancy rate was higher in recipients treated with a 7-day GnRH-based protocol than with PGF alone, because more recipients had a CL on the day of embryo transfer (Baruselli *et al*., 2010). The inclusion of a progestin device in a 7-day GnRH-based protocol in embryo recipients has also resulted in higher pregnancy rates (Beal, 1999). In a field trial involving 1637 recipients treated with GnRH plus a CIDR device and without estrus detection, overall pregnancy rate following embryo transfer to recipients with a CL was 59.9%. The beneficial effects of the J-Synch protocol on fertility has been confirmed recently in a recipient synchronization program (Bó *et al*., 2016).

## Manipulation of ovarian function for superstimulation

The objective of ovarian superstimulatory treatments in cattle is to obtain the maximum number of viable embryos by stimulating growth of antral follicles and ovulation of competent oocytes (Bó and Mapletoft, 2014). Two very important factors influencing variability in superstimulatory response are the intrinsic number of antral follicles in donors, and the stage of follicular development at the time of initiating FSH treatments. Response can be predicted by antral follicle counts done with ultrasonography (Singh *et al*., 2004; Ireland *et al*., 2008), or measurement of circulating concentrations of anti-Müllerian hormone (AMH; *B. taurus*, Rico *et al*., 2009; Ireland *et al*., 2011; *B. indicus*, Batista *et al*., 2014). High antral follicle counts resulted in more ovulations and a greater number of transferable embryos than low antral follicle counts (Ireland *et al*., 2007). Similarly, the top quartile of circulating AMH values was associated with a greater superovulatory response than the lowest quartile (Souza *et al*., 2014).

The conventional protocol of initiating ovarian superstimulation during mid-cycle has been based on anecdotal and experimental evidence suggesting a greater superovulatory response when gonadotropin treatments were initiated between Days 8 and 12 of the cycle (Bó and Mapletoft, 2014). It is now known that mid-cycle is the approximate time of emergence of the second follicular wave (Ginther *et al*., 1989b). However, day of second wave emergence varies between wave types (1 or 2 days later in 2-wave cycles than in 3-wave cycles). In this regard, Nasser *et al*. (1993) showed that superovulatory response was greater when gonadotropin treatments were initiated at the time of follicle wave emergence; 1-day asynchrony reduced the response. The necessity of waiting until mid-cycle to initiate FSH treatment implies monitoring estrus, the obligatory delay and an inability to group donors. An alternative is to superstimulate donors following synchronization of follicular wave emergence (Bó *et al*., 1995; 2002; 2014).

### Follicle ablation

Transvaginal ultrasound-guided follicle ablation followed by FSH treatments 1 or 2 days later is very efficacious (Bergfelt *et al*., 1997), but requires specialized skill and equipment and is difficult to apply in the field. However, follicle aspiration for OPU/IVF will synchronize wave emergence and thus, embryos could be produced both *in vitro* and *in vivo,* from the same donor (Surjus *et al*., 2014).

### Estradiol and progesterone

The preferred approach for synchronization of follicular wave emergence prior to superstimulation is administration of 5 mg E-17β plus 100 mg progesterone and insertion of a progestin device 4 days before initiating FSH treatments. Experimental (Bó *et al*., 1996) and commercial (Bó *et al*., 2002) results have shown that embryo production following this treatment at unknown stages of the estrous cycle is comparable to that initiated 8 to 12 days after observed estrus. By synchronizing follicle wave emergence, the full extent of the estrous cycle is available and the need to detect estrus and wait 8 to 12 days to initiate FSH treatments was eliminated. Although E-17β and its esters are available in most, if not all, countries in South America, this is not the case in many other countries around the world necessitating the use of alternatives to synchronize follicle wave emergence prior to superstimulation.

### Gonadotropin releasing hormone

Attempts to synchronize follicular wave emergence for superstimulation with GnRH were initially unsuccessful; however, subsequent field data were more promising. In these cases, GnRH was administered 1.5 to 3.0 days after the insertion of a progestin device which may have increased the probability of an LH-responsive follicle. Indeed, Bó *et al*. (2008) reported on the strategic use of PGF, a progestin device and GnRH to induce ovulation prior to initiating FSH treatments. Basically, a persistent follicle was induced by treatment with PGF at the time of progestin device insertion; following administration of GnRH 7 days later; ovulation occurred in more than 95% of animals. Superstimulation initiated 36 hours after GnRH (with the progestin device remaining in place) resulted in a superovulatory response that did not differ from controls. More recently, Hinshaw *et al*. (2015) reported no difference in superovulatory response whether GnRH was administered 2 or 7 days after insertion of a progestin device.

## The ruminant reproduction revolution in South America

South American countries are world leaders in beef production, while only Uruguay and possibly Argentina, have dairy industries large enough to exceed local demands. In 2016, Latin American countries had approximately 359 million cattle (accounting for 24% of the world's cattle population) and produced 15.1 million tonnes of beef (Food and Agriculture Organization of the United Nations-FAO, January, 2018). Brazil had 218.2 million cattle and produced 9.3 million tonnes of beef in 2016. Of the main beef-producing countries in South America, Brazil (60.8% of the total), Argentina (14.7%) and Uruguay (3.3%) are also prominent in development and application of assisted reproductive biotechnologies. Through training and continuing education programs, many of which one or more of the authors have organized or participated in, these technologies are being used increasingly in other South American countries. It is through these efforts, in partnership with the pharmaceutical industry, that the ruminant reproductive revolution in South America has become widespread.

It is not possible in this manuscript to discuss all the contributions of South American scientists to the increased and improved understanding of bovine reproduction, especially in *B. indicus* breeds, nor describe in detail the application of reproductive biotechnologies in their breeding herds. However, the reference list in this manuscript (and many other similar publications) provides names of many South American nationals who have made important contributions. Brazil and Argentina, in particular, are very active in research, continuing education and post-graduate student training in reproductive biotechnology, especially in cattle.

Application of assisted reproductive technologies (Sartori *et al*., 2016b), and the state of the embryo transfer (Viana *et al*., 2017) and AI (Baruselli *et al*., 2017) industries in Brazil have been reviewed very recently. Therefore, this paper will focus on Brazil, recognizing that similar advances are taking place in other South American countries, especially Argentina and Uruguay.

**Figure 5:**
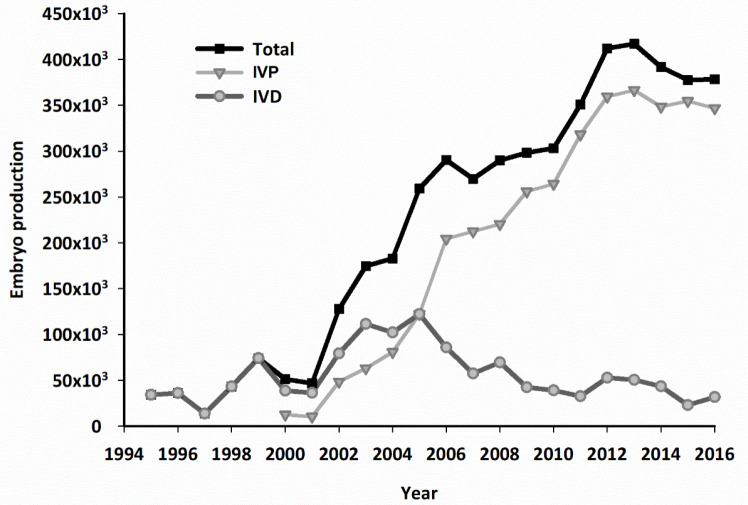
Production of bovine embryos in Brazil from 1995 to 2016. Numbers of embryos produced are shown as Total (*in vivo* and *in vitro* embryos combined); IVP (embryos produced *in vitro*; OPU/IVF); IVD (embryos produced *in vivo;* superovulation and collection). Note that beginning in 2005 numbers of IVP embryos exceeded numbers of IVD embryos and the numbers of IVD embryos began to decline. Complements of Dr. JHM Viana.

The commercial embryo transfer industry began in North America in the early 1970s, and the technology soon spread to South America (Mapletoft, 2013). Although IVD bovine embryo numbers remained modest for several years, Brazil and Argentina consistently ranked in the top five countries outside North America and Europe. Perry (2017) has reported that in 2016 more than 632,000 IVD and 666,000 IVP bovine embryos were produced world-wide. North America accounted for more than 52% of the IVD embryos, but South America accounted for more than 57% of the IVP embryos. The use of IVP in Brazil has increased rapidly since 2000, driven primarily by *B. indicus* breeds which have large numbers of antral follicles from which large numbers of high quality oocytes can be recovered, without superstimulation. Viana *et al*. (2017) reported that embryo transfer accounted for 19.7% of all “Zebu” calves registered in Brazil between 2005 and 2015. *In vitro* embryo production in Brazil increased by 184.0% between 2005 and 2016, while numbers of IVD embryos decreased by 73.7% (Fig. 5; Viana, personal communication, 2017).

Commercial IVP in Brazil has been reported to have gone through three phases (Sartori *et al*., 2016b). The initial phase involved the use of proven donors of high genetic merit in both beef and dairy cattle, and the numbers of IVD and IVP embryos increased similarly. The second phase of growth occurred between 2003 and 2010, driven largely by the need to produce replacement bulls. In 2005, at the peak of this phase, 90.0% of IVP was in beef breeds with Nelore accounting for 82.7% of all embryos. The third phase has involved the use of sex-selected sperm and is associated with a shift in IVP from beef breeds to *B. taurus* dairy breeds. In 2014, IVP in dairy breeds increased by 46.5% (69.0% of the total), exceeding that of beef breeds for the first time.

A similar example of the application of assisted reproductive technologies involves the utilization of FTAI in cattle breeding. Most beef herds in Brazil are composed of *B. indicus* or *B. indicus* crosses, while Argentina and Uruguay tend to have more *B. taurus* breeds and their crosses. It is noteworthy that *B. indicus* breeds tend to have long periods of postpartum anestrus and low body condition scores on pasture, with an increased interval from calving to conception and low fertility (Bó *et al*., 2003). In a pasture-based cow-calf production system, synchronization protocols are necessary to produce pregnancies by AI during a short breeding season and because of problems with estrus detection, FTAI has been incorporated (Bó *et al*., 2013; 2016). Breeding objectives have been to inseminate early in the breeding season followed by early ultrasonographic pregnancy diagnosis and reinsemination of open cattle as soon as possible (Baruselli *et al*., 2017).

Because estradiol preparations have been available in South America, most FTAI protocols include estradiol as a means of synchronizing follicle wave emergence and ovulation (Bó *et al*., 2013). As herd cyclicity and body condition scores are usually low, progestin devices and eCG are usually included in the synchronization protocols (Baruselli *et al*., 2012; Bó *et al*., 2016). To minimize animal handling and the suppressive effect of progesterone on follicle growth, PGF is often administered at the beginning of the protocol, and ECP is administered at the time of progestin device removal (rather than EB 1 day later) with FTAI 48 hours later. Protocols of 6, 7, 8 or 9 days progestin treatment have resulted in similar P/AI, and shortened progestin protocols with a lengthened proestrus involving either estradiol or GnRH have been used with success (Bó *et al*., 2016). The administration of PGF on Day 7 (1 day before progestin removal) in dairy cows has increased P/AI following either FTAI or FTET (Pereira *et al*., 2013). The benefits of eCG in FTET treatment protocols have also been shown in crossbred recipients (Baruselli *et al*., 2010; Bó *et al*., 2012) and high-producing dairy cows (Rodrigues *et al*., 2010).

Resynchronization protocols have also been developed to reduce the interval between FTAI and reinsemination of nonpregnant animals (Bó *et al*., 2016). Traditionally, resynchronization has been done at pregnancy diagnosis 28 to 32 days after FTAI, with a breeding interval of approximately 40 days. Two recent protocols, developed in Brazil, beginning before pregnancy diagnosis (14 or 22 days after the FTAI) have reduced the interval between FTAI and reinsemination to 24 and 32 days, respectively (reviewed in Baruselli *et al*., 2017). The novelty of the 14-day protocol is the use of Doppler ultrasonography 22 days after FTAI for pregnancy diagnosis; a CL area of ≥2 cm^2^ and/or CL blood flow of ≥25% are diagnostic of pregnancy. Resynchronization protocols have led to the adoption of management schemes exclusively for FTAI, eliminating the need for clean-up bulls. In one study, a cumulative pregnancy rate of 87.4% was achieved after three FTAI in a 64-day breeding season, which was greater than achieved with bull exposure after one FTAI (reviewed in Baruselli *et al*., 2017).

**Figure 6:**
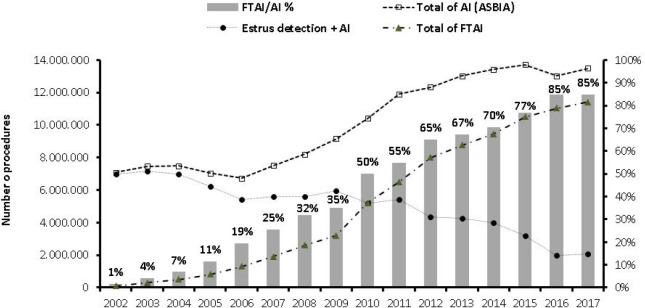
Illustration of the increased use of artificial insemination (AI) and fixed-time AI (FTAI) in Brazil. The numbers of AI were done after estrus detection, while the numbers of FTAI are an estimate based on the number of protocols sold (information provided by pharmaceutical companies in the sector) and the total number of AI is based on the numbers of semen straws sold (*ASBIA, 2017). Data were organized by Departamento de Reprodução Animal-FMVZ-USP, São Paulo, Brazil, 2017. Adapted from Baruselli *et al*., 2017. *ASBIA (Brazilian Artificial Insemination Association).

Data also indicate that FTAI is increasing the use of AI, especially with *B. indicus* sires on *B. indicus* cows in Brazil. There has been more than a 10-fold increase in the use of FTAI in Brazil, from ~1 million protocols in 2005 (11% of all AI) to 10.5 million protocols in 2015 (77% of all AI; Sartori *et al*., 2016b), and a further increase to more than 11 million FTAI in 2016 (Baruselli *et al*., 2017; Fig. 6). Currently, FTAI procedures account for 85% of AI performed in Brazil. In addition, the proportion of dairy cows not inseminated by 70 DIM has decreased resulting in more cows pregnant by 103 DIM and a decrease of approximately 35 days open (Sartori *et al*., 2016b). Industry reports from Argentina and Uruguay for the 2016/17 breeding season indicate a similar trend, especially in beef cattle (3 million and 300,000 FTAI, respectively, representing approximately 10% of their breeding herds). In total, more than 15 million cattle were inseminated by FTAI in these three countries over the past year. Baruselli *et al*. (2017) suggested that the adoption of this technology is an excellent example of a technological change in the production sector emerging from scientific developments in the academic sector.

## Financial benefits of the ruminant reproductive revolution in Brazil

It is estimated that the impact of FTAI on the dairy and beef industries in Brazil is ~U$800 million of extra income per year (Baruselli *et al*., 2017). The use of FTAI has resulted in an estimated 3,500 veterinarians being directly involved with ~U$175 million of economic activity, and there has been an increase of 8% in calf production, representing an additional income of ~U$253 million. Thus, FTAI in Brazil has increased income in the beef production chain by more than a half billion U$ per year. In the dairy industry, FTAI has added U$278 million per year by reducing calving intervals and increasing the use of genetically improved sires. It is estimated that FTAI has reduced the calving interval by 30 days and increased annual milk production by 10%, resulting in ~U$234 million of additional income per year for dairy producers.

## Future considerations for continued development of the embryo transfer industry

Despite the considerable progress made in the application of bovine embryo transfer technologies in South America over the past two decades, especially with IVP in Brazil, donor selection has leaned heavily on phenotypic traits. For real genetic gain, generation intervals must be shortened, selection intensity must be increased and the accuracy of selection must be improved (Smith 1988). Genomic techniques have now become essential for the selection of donors (male and female) used in embryo transfer (Ponsart *et al*. 2014). In the dairy industry, genetic progress has been accelerated by the use of embryo transfer combined with genomic selection. This could have an even greater impact in the beef industry, as genomic traits for beef production are further developed.

Although the use IVP embryos in South America has increased greatly over the past 15 years, a major limitation has been that most IVP embryos had to be transferred fresh. Cryopreservation of IVP embryos, especially within *B. indicus* breeds, has been very difficult. Although improvements in culture conditions and embryo grading systems will likely increase success with cryopreservation, a recent publication by Sanches *et al*. (2016) provides convincing evidence that the Direct Transfer of frozen/thawed IVP embryos can result in satisfactory pregnancy rates. With continued improvements in this technology, the use of cryopreserved IVP embryos will no doubt increase.

Sex-selected sperm in the production of IVP embryos has obvious advantages, especially with the recent increase in the use of IVP in the generation of dairy embryos. However, Siqueira *et al*. (2017) have recently reported that the use of reverse X-sorted spermatozoa in the production of IVP embryos resulted in an alteration of embryonic programming that persisted postnatally and caused an effect on milk production in adulthood. Thus, benefits of the use of sex-selected semen in the production of IVP embryos could be offset by adverse programming events. It is not known whether regular sexing technologies have similar effects, and whether this effect can be overcome. Obviously, this finding raises concerns that must be resolved.

## Summary and conclusions

The ability to control follicular wave emergence and ovulation in cattle has eliminated the need for estrus detection allowing for FTAI and FTET, and the initiation of superstimulation of embryo donors on predetermined schedules. In addition, the utilization of eCG in these protocols has increased pregnancy rates, especially in cattle experiencing postpartum anestrus. Practitioners in most South American countries are utilizing these protocols which has facilitated the application of assisted reproductive technologies. The utilization of FTAI has resulted in great increases in the use of AI, and the adaption of these protocols for FTET has increased the use of bovine embryo transfer, especially with IVP embryos. Currently, FTAI procedures account for 85% of all AI done in Brazil, and a similar trend is occurring in Argentina and Uruguay. Although the numbers of IVD embryos has tended to decrease, especially in Brazil, IVP has increased greatly with more than 57% of all IVP embryos in the world produced in South America. There has been a remarkable shift in the use of IVP from beef breeds to dairy breeds in Brazil, with only a modest decrease in embryo production. Collectively, the successful application of assisted reproductive technologies in South America is resulting in the dissemination of new and improved genetics and increased reproductive performance in all classes of cattle, with a corresponding increase in economic activity.
